# Genetic diversity of honeybee colonies predicts gut bacterial diversity of individual colony members

**DOI:** 10.1111/1462-2920.16150

**Published:** 2022-08-12

**Authors:** Calum Bridson, Latha Vellaniparambil, Rachel E. Antwis, Werner Müller, R. Tucker Gilman, Jennifer K. Rowntree

**Affiliations:** ^1^ Faculty of Science and Engineering University of Manchester Manchester UK; ^2^ Faculty of Biology Medicine and Health, Lydia Becker Institute of Immunology and Inflammation University of Manchester Manchester UK; ^3^ Department of Infectious Diseases, Medical Microbiology and Hygiene University of Heidelberg Heidelberg Germany; ^4^ Translational Lung Research Centre (TLRC) Heidelberg Germany; ^5^ Ecology and Environment Research Centre, Department of Natural Sciences Manchester Metropolitan University Manchester UK; ^6^ School of Science, Engineering and Environment University of Salford Salford UK; ^7^ Miltenyi Biotec, Bergisch Gladbach Germany; ^8^ School of Biological and Marine Sciences University of Plymouth Plymouth UK

## Abstract

The gut microbiota of social bees is relatively simple and dominated by a set of core taxa found consistently in individuals around the world. Yet, variation remains and can affect host health. We characterized individual‐ and regional‐scale variation in honeybee (*Apis mellifera*) gut microbiota from 64 colonies in North‐West England by sequencing the V4 region of the 16S rRNA gene and asked whether microbiota were influenced by host genotype and landscape composition. We also characterized the genotypes of individual bees and the land cover surrounding each colony. The literature‐defined core taxa dominated across the region despite the varied environments. However, there was variation in the relative abundance of core taxa, and colony membership explained much of this variation. Individuals from more genetically diverse colonies had more diverse microbiotas, but individual genetic diversity did not influence gut microbial diversity. There were weak trends for colonies in more similar landscapes to have more similar microbiota, and for bees from more urban landscapes to have less diverse microbiota. To our knowledge, this is the first report for any species that the gut bacterial communities of individuals are influenced by the genotypes of others in the population.

## INTRODUCTION

Microbial symbionts are ubiquitous in their association with animals, and their influence on host species ranges across a spectrum from deleterious to beneficial (Ferrari & Vavre, 2011). An important class of symbionts resides in the gut. Gut microbial communities can affect host health (Clemente et al., 2012; Round & Mazmanian, 2009), ecology, and evolution (Engel & Moran, 2013; McFall‐Ngai et al., 2013), for example, by influencing host behaviour (Cryan & Dinan, 2012), protecting against parasites (Koch & Schmid‐Hempel, 2011a), or providing insecticide resistance (Kikuchi et al., 2012).

Social bees provide a good model system for studying gut microbiota (Zheng et al., 2018). The bee gut microbiota is relatively simple and is dominated by a core of nine bacterial phylotypes that are found consistently across continents, genotypes, and landscapes (Moran, 2015; Moran et al., 2012). These phylotypes include six Proteobacteria [*Giliamella apicola*, *Snodgrassella alvi*; Kwong & Moran, 2013; *Frischella perrara*; Engel, Kwong, & Moran, 2013; *Bartonella apis* Kešnerová et al., 2016; Alpha 2.1 (*Commensalibacter*), and Alpha 2.2 (*Parasaccharibacter apium*); Martinson et al., 2011; Corby‐Harris et al., 2014; Corby‐Harris & Anderson, 2018], two Firmicutes [*Bombilactobacillus* spp. (formerly *Lactobacillus* Firm‐4) and *Lactobacillus* Firm‐5; Killer et al., 2014; Olofsson et al., 2014; Zheng et al., 2020], and an Actinobacterium (*Bifidobacteria asteroides*; Bottacini et al., 2012).

Despite the consistent presence of these phylotypes in the bee gut bacterial community, there is variation in the relative abundance of the core phylotypes and in the core strains present (Engel et al., 2012; Moran et al., 2012; Powell et al., 2016). Variation in bacterial composition can lead to functional differences (Engel et al., 2012) in traits such as pollen and saccharide breakdown (Engel et al., 2012; Lee et al., 2015) and resistance to disease (Koch & Schmid‐Hempel, 2012). Until now studies have focused on the differences among regions and continents, but there is little knowledge of how the bee microbiota composition varies within regions that comprise multiple apiaries across a landscape. At this scale, the set of apiaries can be considered a population or metapopulation, with the potential for genetic material to be transferred among them. Understanding variation in the microbiota at the regional level is important because this variation can influence how host populations respond to change and their resilience to parasites and pesticides (Hehemann et al., 2012; Koch & Schmid‐Hempel, 2012; Zilber‐Rosenberg & Rosenberg, 2008). Furthermore, if individual colonies have distinct microbiotas, then this may drive differences among colonies in fitness and health, and thus could be important for understanding and managing bee declines or optimizing honey production (Ribière et al., 2019).

Studying multiple colonies across a region can help us to understand the variables that influence the honeybee microbiota, for example, the effect of different land use types on the honeybee gut microbial community (Engel et al., 2016). Land use can be a proxy for floral composition (Kleijn & Van Langevelde, 2006) and potentially for other environmental factors such as exposure to pollutants (Botías et al., 2017). Land‐use change is also thought to be a major factor in bee decline (Potts et al., 2010), and land‐use per se has been shown to influence gut microbiota composition in other systems (Teyssier et al., 2018). Exploratory work has found a link between land use and the bee bread microbiome (Donkersley et al., 2018), but there is little knowledge of how land use affects the bee gut microbiota. Host genotype has also been shown to shape host microbiotas in some systems (Griffiths et al., 2019; McKnite et al., 2012; Zoetendal et al., 2001). This may be particularly important for honeybee colonies because the majority of workers in a colony are related to the queen, although as honeybee queens are promiscuous, workers are not always full siblings (Estoup et al., 1994). Furthermore, haplodiploidy in honeybees means full sibling workers share 75% of their genes on average.

**FIGURE 1:**
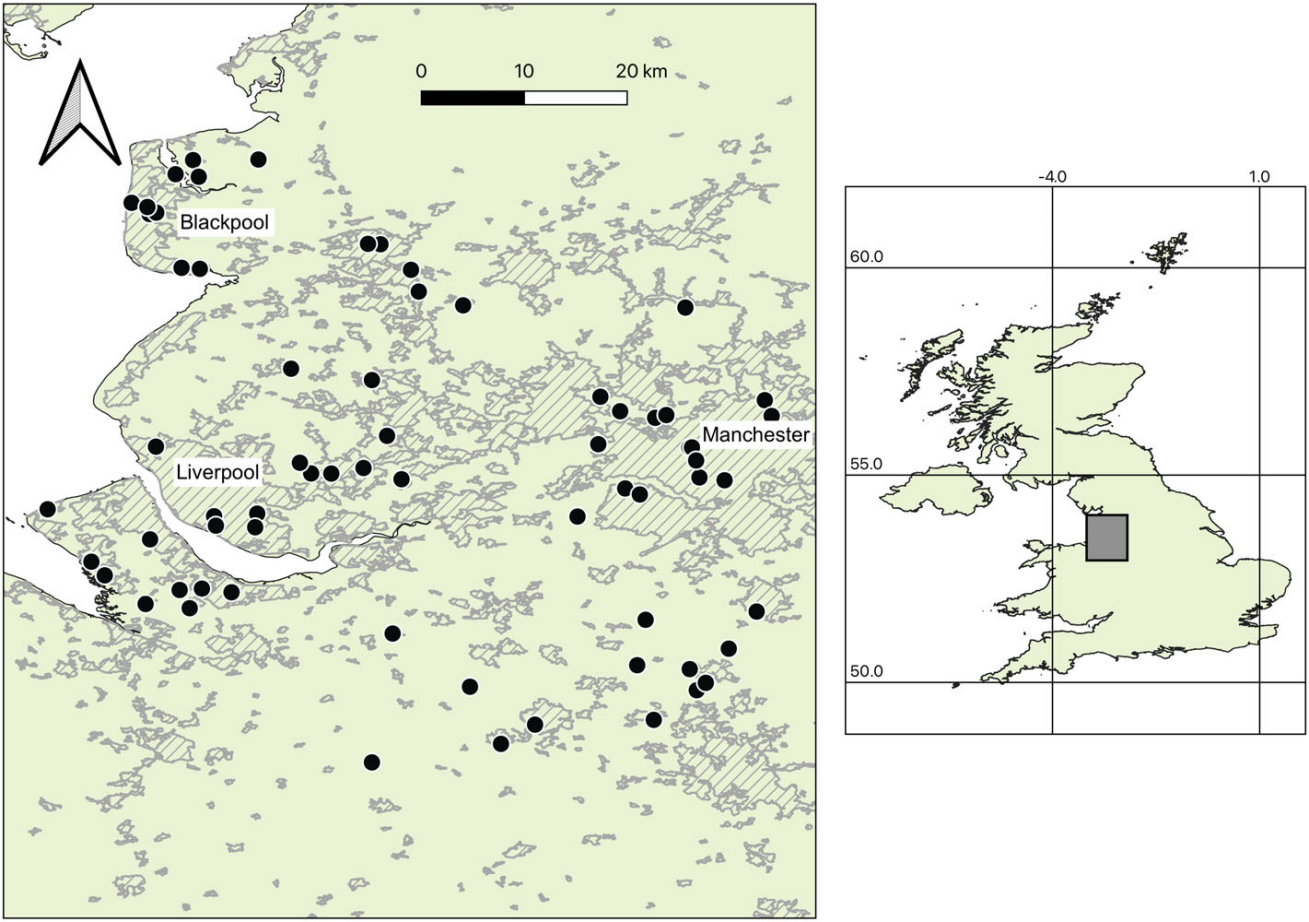
Map of the study area in North West England with location shown as a grey box on the inset map of the United Kingdom. Colonies are shown as black dots and urban areas as hatched grey. The UK layer was downloaded from map.igismap.com and the urban layer (showing built areas in December 2011) from geoportal1‐ons.opendata.arcgis.com.

In this study, we sampled gut bacterial communities of multiple individuals from 64 honeybee colonies across North‐West England, UK, providing high landscape coverage (Figure 1). We used Illumina V2 MiSeq sequencing to the V4 region of the 16SrRNA gene to characterize each individual's gut bacterial community. There were four main aims of the study: (i) to describe the gut bacterial community composition of honeybees in North‐West England; (ii) to investigate how variation in the bacterial community is partitioned among colonies; and (iii) to determine whether landscape diversity and composition or (iv) the genetic diversity of colonies or individuals influences the honeybee gut bacterial community.

## EXPERIMENTAL PROCEDURES

### Sample collection

We sampled individual honeybees from 64 colonies across North West England (Figure 1) in May and June 2016. The colonies were spread across both rural and urban environments, including three major urban centres, and were mostly owned by amateur beekeepers who volunteered to participate in the study. We sampled one colony per apiary, and five randomly selected nurse bees per colony. Nurse bees are adult workers that have yet to leave the nest, and that perform roles such as feeding the brood and guarding the nest (Winston, 1987). We sampled the nurse stage because these bees take food from multiple foragers and distribute it to the larvae. Hence, they are exposed to a wide variety of food sources, and their microbiota may better reflect that of the colony as a whole (Kapheim et al., 2015). We identified nurse bees by shaking a brood frame into a bucket and collecting only those bees that did not fly away. After collection, bees were euthanized in 100% ethanol and frozen at −20°C until further use (Koch & Schmid‐Hempel, 2011b; Powell et al., 2014).

We were unable to standardize the age of the nurse bees that we sampled. The behavioural role of worker bees has been shown to influence gut microbiota composition (Jones, Fruciano, Marchant, et al., 2018), but it is unclear whether or how the microbiota varies with age within a specific role. Variability in age could potentially increase variability and diversity of the microbiota within a colony, but it is unlikely to explain the patterns we report in the results.

### Dissection and DNA extraction

Bees were washed in 1% (v/v) sodium hypochlorite solution for 5 min, followed by three washes in autoclaved MilliQ water, to remove any external bacteria that could contaminate the sample. To further reduce contamination, we carried out dissection, DNA extraction, and other molecular procedures in a PCR hood (UVP, CA) in which all equipment and surfaces had been UV‐sterilized for 30 min. The midgut and hindgut were dissected using sterile instruments, with one gut tract used per sample. We carried out negative extractions each day to monitor any contamination, which equated to two or three colonies per negative extraction. All negative extraction samples were processed as normal, but instead of adding honeybee guts, sterilized forceps were scraped against the inside of the Eppendorf.

We placed dissected guts in sterile 2‐ml Eppendorfs with 180 μl SDS lysis buffer (200 mM NaCl, 200 mM Tris, 20 mM EDTA, 6% SDS; Moran et al., 2012). To homogenize the guts, we added a 5‐mm stainless steel bead (Qiagen, UK) and 500 μl of 0.1 mm acid‐washed glass beads (Sigma‐Aldrich, UK) to the sample, then shook the sample twice for 2.5 min at 30 Hz on a Mixer Mill, inverting samples between shakings (Retsch, Germany; Engel, James, et al., 2013). After bead‐beating, we added of 30 μl of 10 mg/ml lysozyme (Sigma‐Aldrich) and incubated the samples at 37°C for 1 h to digest Gram‐positive cell walls (Teng et al., 2018). We extracted DNA using the Qiagen DNeasy Blood and Tissue Kit following the protocol for Gram‐positive bacteria from step 4 of the DNeasy handbook (Qiagen DNeasy Blood and Tissue Handbook, 2006). The only modification to this protocol was to incubate the samples in proteinase K and buffer AL for 1 h at 56°C after the lysozyme step. The DNA was eluted in 200 μl of buffer AE and stored at −20°C until further use.

### Gut bacterial community sequencing

We sequenced the gut bacterial communities using a modification of the dual‐index sequencing protocol described by Kozich et al. (2013) and Antwis et al. (2018). Briefly, the hypervariable V4 region of the 16S rRNA gene was amplified using the universal primers 515F (TATGGTAATTGTGTGCCAGCMGCCGCGGTAA) and 806R (AGTCAGTCAGCCGGACTACHVGGGTWTCTAAT), modified with Illumina adaptors and index sequences. The V4 region was amplified in triplicate for each sample in a single PCR step using the following conditions: 95°C for 15 min; 25 cycles of 95°C for 20 s, 55°C for 60 s, 72°C for 60 s; and then 72°C for 10 min. The triplicate PCRs were pooled, and the amplicons were cleaned using Agencourt Ampure beads (Beckman Coulter Genomics, Indianapolis, IN, USA) and quantified and quality checked using a Tapestation (Agilent Technologies Inc., CA, USA). DNA concentrations were determined using Qubit (Thermo Fisher Scientific, USA). We removed 29 samples because they had insufficient concentrations of DNA, leaving two to five samples (i.e. bees) per colony. Equimolar amounts of each remaining sample were pooled and sequenced together, along with 10% PhiX v3 (Illumina, San Diego, CA, USA) as an in‐run control. Sequencing was conducted using Illumina v2 chemistry (2 × 250 bp paired‐end sequencing) on the MiSeq platform (Illumina) at the University of Salford.

### Bioinformatics

We processed the sequence data using the Dada2 v1.6.0 pipeline (Callahan et al., 2016) in R 3.6.1 (R Core Team, 2017). We used default parameters for all functions, except we trimmed the forward reads at 240 bp, the reverse reads at 230 bp and we removed sequences when the maximum expected error (maxEE) was greater than two. We also removed 36 amplicon sequence variants (ASVs) for having lengths greater than 236 bp. Taxonomic assignment of the remaining sequences was performed using the RDP Naïve Bayesian Classifier method described by Wang et al. (2007), with training sets constructed from the Silva v128 database (Quast et al., 2012). We removed ASVs that were not taxonomically assigned to the kingdom ‘Bacteria’ or that were classified to the class ‘Chloroplast’ or the family ‘Mitochondria’. At this point, all remaining ASVs were assigned down to at least the phylum level. We removed ASVs that were only present in one sample, as these are more likely to be erroneous (Goodrich et al., 2014). The 52 negative controls contained very low levels of contamination, with the total reads in each negative control ranging from 7 to 118. Across the negative controls, there were 18 ASVs that were also found in the true samples. We removed ASVs if the mean number of reads across all true samples was not at least 10× higher than in the negative controls. This led to the removal of seven ASVs, all of which were known contaminants from previous studies (Salter et al., 2014). The other 11 ASVs in the negative controls were all members of the previously identified core phylotypes and were the most abundant ASVs found in this study. After all filtering, 136 of the original 1279 ASVs remained in our analyses. We generated rarefaction curves for each sample using the command ‘rarecurve’ in the package vegan (Oksanen et al., 2018; Supplementary Figure S1). We applied maximum likelihood analysis to construct a phylogenetic tree of the filtered ASVs using the phangorn package v2.3.1 (Schliep, 2011) with a GTR + G + I nucleotide substitution model.

### Landscape analysis

We determined the landscape composition surrounding each colony (except one for which the GPS location was not recorded) using data from the Land Cover Map 2015 (25 m raster, GB; Rowland et al., 2017). The Land Cover Map classifies the land cover of each 25 m^2^ pixel into one of 21 land cover classes. We used QGIS version 2.18.14 (QGIS Development Team, 2017) to overlay the Land Cover Map raster layer onto the locations of colonies and to define buffer zones around each colony at 500 m, 1.5 km, 5 km and 10 km radii. Buffer zone sizes were chosen based on the frequency at which honeybees forage at different distances from the colony (Steffan‐Dewenter & Kuhn, 2003; Visscher & Seeley, 1982; Waddington et al., 1994). We used the LecoS plugin (Jung, 2016) for QGIS to determine the proportion of each buffer zone that belonged to each of the 21 land cover classes. The landscape diversity of each buffer zone around each colony was determined by estimating the Hill number (*q* = 1) from the raw land‐use proportion data using the package hillR (Li, 2018). The suitability of Hill numbers to estimate landscape diversity is supported by the fact that the related Shannon index is widely used to characterize landscape diversity (Nagendra, 2002; Ramezani, 2012).

### Genotyping

We determined individual bee genotypes at five microsatellite loci according to the protocol described by Evans et al. (2013) using the primers detailed in Supplementary Table S7. These microsatellite loci have been used in other honeybee population genetic studies (Muñoz et al., 2009, 2012). We ran the microsatellites as two separate multiplexes, with primers A113, AP043, and AP055 in multiplex 1 and primers A007 and B124 in multiplex 2, using the Qiagen Type‐It master mix kit (Qiagen, Valencia, CA) in a final reaction volume of 10 μl. PCR amplification involved denaturation at 94°C for 5 min, followed by 30 cycles of 95°C for 30 s, 57°C for 30 s, and 72°C for 30 s, with a final elongation step at 72°C for 30 min. The size of the microsatellites was determined by capillary electrophoresis on an ABI 3730 DNA Analyser (Applied Biosystems, CA, USA) at the University of Manchester Sequencing Facility, using the GeneScan™ LIZ500 (Thermo Fisher Scientific, USA) size standard. The output peaks were scored using Genemapper v5.0 software (Applied Biosystems) and sorted into allele bins using the MsatAllele package v1.05 (Alberto, 2009) in R (R Core Team, 2017). A subset of samples were repeated to ensure consistency of allele calling. The function hw.test in the package pegas (Paradis, 2010) was used to test whether the alleles were in Hardy–Weinberg equilibrium.

### Statistical analyses

We measured α‐diversity using Hill numbers with q values of 0, 1 and 2, which differ in how they weight rare ASVs (Hill, 1973). When *q* = 0 the Hill number reports absolute species richness, and when *q* > 0 the Hill number increases as the combination of richness and evenness of the community increases. For individual bees, we calculated Hill numbers from the raw read counts using rarefaction/extrapolation curves to account for differences in sequencing depth among samples (Chao et al., 2014). For colonies, we estimated Hill numbers from the mean proportional abundance of ASVs across all samples from the colony.

We calculated β‐diversity from the ASV abundance data. Due to unequal sequencing depth across samples, we used cumulative sum scaling (CSS; Paulson et al., 2013) to produce a normalized matrix of ASV abundances. We generated CSS‐normalized ASV abundance matrices for both individual bees and colonies. Abundance at the colony level was calculated by summing the normalized abundance values for each ASV across all samples from a colony and then dividing by the number of samples from that colony. We used weighted UniFrac distances to produce pairwise dissimilarity matrices from the CSS‐normalized abundance data at the individual and colony levels by applying the ‘distance’ function in phyloseq 1.21.0 (McMurdie & Holmes, 2013). The dissimilarity matrices were visualized with non‐metric multidimensional scaling (nMDS) performed by the ‘ordinate’ function in phyloseq.

We partitioned variation in the gut bacterial community within and among colonies using permutational multivariate analysis of variance (PERMANOVA; Anderson, 2001) with 9999 permutations applied to our weighted UniFrac dissimilarity matrices and implemented in the ‘adonis’ function of the package vegan (Oksanen et al., 2018). We used a K‐nearest neighbour (KNN) graph‐based analysis of the CSS‐normalized individual‐level data, implemented with the functions ‘phyloseqGraphTest’ and ‘igraph’ (Csárdi & Nepusz, 2006), to ask whether the individuals with the most similar microbiotas were likely to be nestmates or from different colonies. We performed the KNN analysis for *K* = 3, because individuals from most colonies had at least three nestmates that were sampled.

We used Mantel and partial Mantel tests (9999 permutations; Mantel, 1967) to investigate the relationship of individual and colony bacterial composition with the geographic proximity of the colonies, the landscape composition around the colonies, and the individual and colony genotype. The bacterial community composition distance matrices were created at the individual level for testing the relationship with individual genotype and at the colony level for testing the relationship with proximity of the colonies, landscape composition and colony genotype. The geographic distance matrix was created by converting the longitude and latitude coordinates of each colony into Universal Transverse Mercator coordinates using the R package rgdal (Bivand et al., 2020). Distance matrices for land use composition were generated for each of the four buffer zones using Bray–Curtis dissimilarities calculated with the function ‘vegdist’ in the package vegan (Oksanen et al., 2018) and we performed partial Mantel tests for each buffer zone separately. Genetic dissimilarity matrices were generated from the individual and colony‐level multi‐locus allele data, using Roger's genetic distances (Rogers, 1972) in the package adegenet (Jombart, 2008). To account for potential spatial autocorrelation in the colony‐level analyses, we included the geographic distance matrix.

To understand the factors that predict the diversity of the gut microbiome at the colony level, we regressed the colony‐level Hill numbers on (i) the mean proportion of alleles shared between individuals in the colony, (ii) the mean heterozygosity of the colony, (iii) the landscape diversity in the 5 km buffer zone around the colony, (iv) the proportion of urban land surrounding the colony, and the (v) northing and (vi) easting of each colony. We included the number of bees sampled from the colony as a categorical predictor in the model. This controls for the fact that we expect to find more ASVs in colonies from which more bees were sampled but makes no assumptions about the rate at which ASVs accumulate as the number of samples increases. We weighted the variance of each colony‐level data point in proportion to the square root of the number of samples (i.e. individual bees) from which that data point was calculated. Initially, we fitted linear regressions using the package nlme (Pinheiro et al., 2020) which allowed us to include spatially autocorrelated error in the model. However, we found no evidence for spatial autocorrelation, and therefore we removed spatial autocorrelation from the model and fitted linear regressions using the function lm in base R. For each Hill number (i.e. q ∈ {0, 1, 2}), we fitted models that included each possible combination of the predictors in the full model. We weighted each model according to its Akaike weight and calculated the effect size of each predictor as the weighted average across all models in which that predictor appeared (Burnham & Anderson, 2002). To obtain *p* values for each predictor, we computed the confidence distribution around the effect size of the predictor in each model in which it appeared, weighted these according to the Akaike weights of the models, and summed across all models. The *p* value associated with the predictor is two times the minimum of the proportion of the confidence distribution that is greater than zero or is less than zero (Gilman et al., 2018).

To understand the factors that predict the diversity of the gut microbiome of individuals within colonies, we regressed the individual‐level Hill numbers on the heterozygosity of the individual and on all six colony‐level predictors. We included a random effect of colony in the model, and fitted models by maximum likelihood using the package lme4 (Bates et al., 2015). For each Hill number (i.e. q ∈ {0, 1, 2}), we calculated the effect size and *p* values associated with each predictor as described for the colony‐level analysis.

## RESULTS

A total of 6,900,477 reads passed the quality filter thresholds, from an initial output of 9,487,873 reads from the MiSeq run. The number of reads per sample ranged from 7758 to 44,343 (mean 23,551), with a total of 136 ASVs detected across the study. The rarefaction curves for each sample reached a plateau (Supplementary Figure S1). Thus, the sequencing depth was sufficient to characterize the bacterial communities of the samples.

### Gut bacterial community composition

In line with previous studies, 98.9% of reads were members of the literature‐defined core taxa, and only six samples had bacterial community compositions that consisted of less than 90% core phylotypes. All core phylotypes except *Parasaccharibacter apium* were found in this study.

Each core taxon present in this study was represented by multiple ASVs, but the majority of ASVs were rare (Supplementary Table S1). The 20 most abundant ASVs comprised 94.5% of all reads in the study. These included representatives from each of the nine core phylotypes except *P. apium* and also included the non‐core species *Lactobacillus kunkeei*. Thus, the pattern of dominance by the core phylotypes is driven by just a one or two ASVs from each taxon. The most abundant core phylotypes in this region were *Lactobacillus* Firm 5 and *Gilliamella apicola*, which together constituted 48.6% of the regional honeybee gut bacterial community (Supplementary Table S1). Outside of the core, the majority of ASVs belong to the family *Enterobacteriacae*.

All of the core phylotypes found in this study were present in each of the colonies (Supplementary Table S2). In contrast, only 67% of individuals possessed all of the core phylotypes, and 7.8% of individuals were missing two or more phylotypes. However, only the *Commensalibacter* phylotype was detected in less than 90% of individuals.

### Partitioning gut bacterial community variation

**FIGURE 2:**
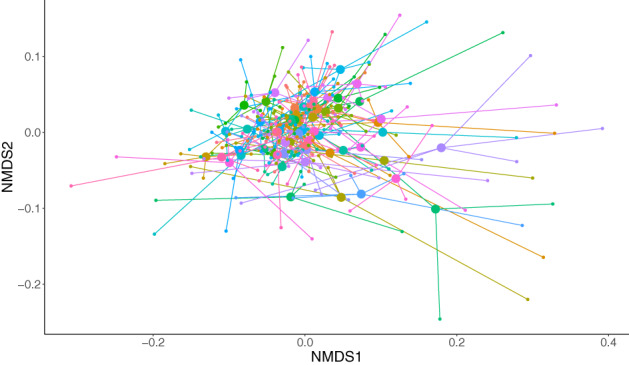
Non‐metric multidimensional scaling (nMDS) ordination plot showing the variation among individual honeybee gut bacterial communities, and among the mean colony bacterial communities, across North West England. Large circles are the centroid bacterial community for each colony, which are connected by lines to each individual (smaller circles) from that colony.

Colony membership had a significant effect on honeybee gut bacterial community composition, explaining 41% of the variation among samples (PERMANOVA: *R* ^2^ = 0.414, df = 63, 229, *p* = 0.0001; Figure 2). There were marginally significant differences in sample variance within colonies (betadisper: *F* = 1.38, *p* = 0.051), but PERMANOVA is robust to differences in variance when designs are well balanced (Anderson & Walsh, 2013). Due to the differences among colonies in sample size, we randomly selected three individuals from each colony to ensure there was an equal number of observations per colony and performed the PERMANOVA on this subset of the data, using 9999 permutations as before. We repeated the analysis 50 times, with each analysis producing the same *p* value. A *k*‐nearest neighbour (KNN) analysis confirmed that the gut bacterial communities of bees from the same colony were more similar than expected by chance (*p* = 0.001). However, only 5.7% of edges were between samples from the same colonies. Thus, while there is structure by colony, high variation within colonies means that bees from the same colony did not have distinguishable colony‐specific microbiota.

### Landscape effects

We found no evidence that colonies that were closer together geographically possessed more similar gut microbial communities (Mantel test: *r* = 0.020, *p* = 0.336). There were marginally significant effects of landscape composition on the pooled gut bacterial community composition of the colonies at the 5‐km scale (Partial Mantel test: *r* = 0.095, *p* = 0.057), and 10‐km (Partial Mantel test: *r* = 0.073, *p* = 0.087) scales. There was no significant effect of landscape composition on colony pooled gut microbial composition at the 500‐m (Partial Mantel test: *r* = 0.041, *p* = 0.234) or the 1.5‐km scales (Partial Mantel test: *r* = 0.072, *p* = 0.109).

### Genetic effects

There were 81 alleles across the five microsatellite loci we studied, with the number of alleles per locus ranging from nine to 22 (Supplementary Table S3). Only 0.14% of the data were null alleles. Of 293 individual bees, only 14 did not have unique genotypes. Thus, the five loci we studied were enough to discriminate among most individuals. The alleles AP043 (*p* = 0.106) and A007 (*p* = 0.772) were in Hardy–Weinberg equilibrium, while the alleles A113 (*p* = 0.036), AP055 (*p* < 0.001) and B124 (*p* < 0.001) were not. The genetic distances among individuals are shown in Supplementary Figure S2, and the allelic richness and heterozygosity data for each colony are reported in Supplementary Table S4. Individuals and colonies with more similar genotypes did not have more similar bacterial communities (individuals, Mantel test: *r* = −0.042, *p* = 0.928; colonies, Mantel test: *r* = −0.137, *p* = 0.979).

### Alpha diversity

**FIGURE 3:**
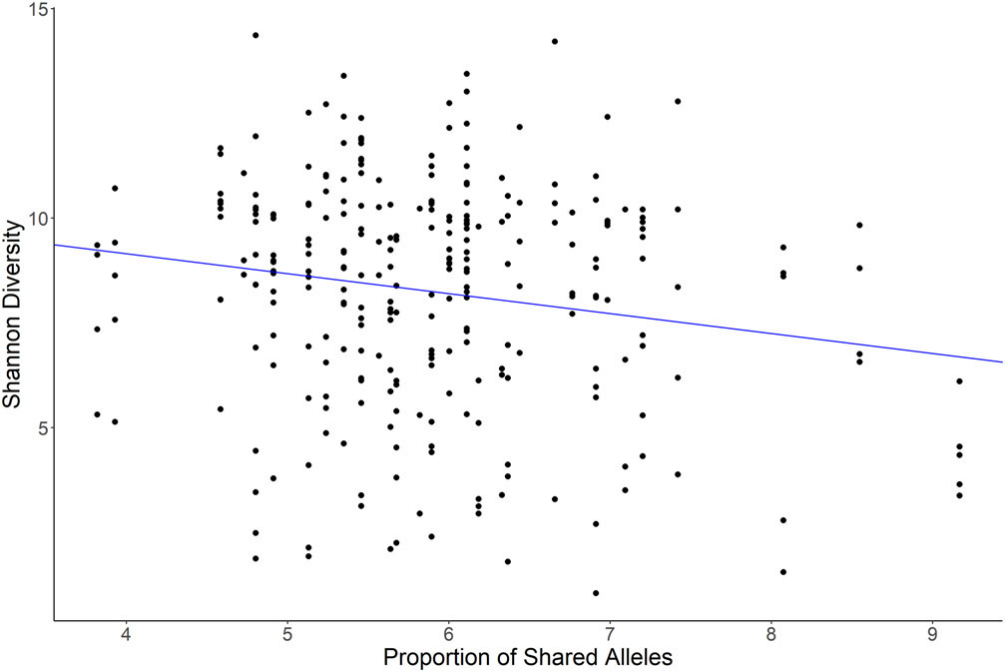
Relationship between proportion of alleles shared among individuals in a colony and Shannon diversity (Hill number *q* = 1) of those individuals' gut bacterial communities. Lower shared alleles indicate higher genetic diversity at the colony level. Therefore, in colonies that are more genetically diverse, individuals had more diverse gut bacteria. The regression line (slope = −0.47, *p* = 0.040) is the line of best fit to the data, weighted across all fitted models.

We found a marginally significant relationship between the species richness of colony pooled gut bacterial communities and the proportion of urban land surrounding the colony (*p* = 0.062; Supplementary Table S5). The least urban colonies had approximately 3.7 more species in their pooled gut microbiota than the most urban colonies. We found a marginally significant relationship (*p* = 0.095) between the proportion of alleles shared between pairs of colony‐mates and the biodiversity of the gut microbiome at the colony level for *q* = 1 (i.e transformed Shannon diversity) and a non‐significant trend in the same direction (*p* = 0.101) for *q* = 2 (i.e. transformed Simpson diversity). In both cases, colonies with lower proportions of shared alleles (i.e. higher genetic diversity) had more diverse gut bacterial communities. At the individual level, bees from colonies with fewer alleles shared between pairs of workers (i.e. higher genetic diversity) had greater gut bacterial biodiversity when *q* = 1 (*p* = 0.040) and when *q* = 2 (*p* = 0.040) (Figure 3, Supplementary Table S6).

## DISCUSSION

We report a detailed investigation of honeybee gut bacterial communities across a geographic region. A few core bacterial phylotypes dominated the communities, and each phylotype was in turn dominated by one or two ASVs. There was structuring of the microbiota by colony, but an individual's colony membership could not be predicted from its microbiota composition. We found weak evidence that more urban colonies had less diverse gut bacterial communities, and that colonies from more similar environments had more similar gut bacterial communities. We found stronger evidence that individual bees had greater gut bacterial diversity when their colonies had greater genetic diversity. This last result means that, in honeybees, the genotypes of individuals predict the bacterial communities of other individuals in the population. To our knowledge, this is the first time this has been shown for any species.

### Gut bacterial community composition

We provide further evidence that the honeybee gut bacterial community is dominated by a core set of phylotypes that have been found consistently across landscapes and continents (Moran, 2015; Moran et al., 2012). The only absentee from the literature‐defined core was *Parasaccharibacter apium*. The absence of *P. apium* is not surprising as it is generally not found in worker guts, and instead can be found in worker hypopharyngeal glands, the guts of mature queens, larvae and the colony environment (Corby‐Harris et al., 2016). A lack of *P. apium* in our data suggests our methods successfully limited contamination from bacteria in the colony environment and the worker crop.

The literature‐defined core phylotypes comprised a similar proportion of the honeybee gut bacterial community in this study and in previous studies (Moran, 2015). Furthermore, *Lactobacillus* Firm 5 and *Gilliamella apicola* were the most abundant phylotypes in the honeybee gut here, and both have been found to dominate in other studies (Martinson et al., 2012; Moran et al., 2012). Therefore, our results suggest that the bacterial communities of honeybee guts in North West England are similar to those seen in other places.

Within each core phylotype, there were multiple ASVs, but most of the reads within each belonged to just one or two ASVs. Previous studies have found multiple strains of a phylotype within a single individual, with one strain often dominating (Moran et al., 2012). The amplicon length used in this study precludes the discrimination of different strains, but the study extends the conclusion that the same ASVs dominate not just within individuals, but across the region, among different host genotypes, and in bees exposed to different land use types.

### Landscape effects

In the 5 km and 10 km buffer zones, there was a trend for landscapes with more similar land use composition to harbour colonies with more similar gut bacterial communities. These results are consistent with a small body of work that links honeybee microbiota to land use. Jones, Fruciano, Hildebrand, et al. (2018) found small differences among the gut microbiota of colonies placed near oil seed rape fields and those placed further away. Donkersley et al. (2018) found that land‐use surrounding colonies predicts the species richness of the bee bread microbiota. In contrast, in three bumblebee species, Cariveau et al. (2014) found no difference in the microbiota of individuals collected at an active or at an abandoned cranberry farm.

We found a weak trend linking the diversity of the honeybee gut bacterial community to urbanization. In more urban environments, gut bacterial communities had lower species richness. Among synanthropic bird species, similar patterns have been reported in house sparrows (*Passer domesticus*; Teyssier et al., 2018) in Belgium and herring gulls (*Larus argentatus*; Fuirst et al., 2018) in New York, but the opposite pattern was reported in white‐crowned sparrows (*Zonotrichia leucophrys*; Phillips et al., 2018) in California. Bosmans et al. (2018) reported higher gut bacterial diversity in bumblebee (*Bombus terrestris*) queens from two forested sites than from three urban sites in Belgium, but with only five sites sampled it was impossible to attribute differences with confidence to urbanization.

### Genetic effects

We found individual bees from more genetically diverse colonies had more diverse gut bacterial communities. In a study in which colony genetic diversity was experimentally manipulated, Mattila et al. (2012) found that colonies with more genetic diversity had more diverse bacterial communities. Our results advance those of Mattila and colleagues in two important ways. First, the genetic diversity in our study was not manipulated, so our results show that naturally occurring differences in genetic diversity are sufficient to predict the diversity of gut bacterial communities. Second, the differences in bacterial diversity uncovered by Matilla and colleagues appeared at the level of the colony. Such differences could occur in two non‐exclusive ways. First, that bees with different genotypes could host different bacterial communities. Individual bees in genetically diverse colonies might not host more diverse communities themselves, but if each bee hosts a different set of bacteria, then the pooled community at the colony level would be more diverse. Second, in more genetically diverse colonies, individual bees host more diverse bacterial communities. In our study, the latter explanation was true. This shows that the genotype of each individual in the colony predicts the microbiome of other individuals.

Our results do not show that the genetic diversity at the colony level *causes* diversity in the individual gut bacterial community. For example, it might be true that colonies in areas with a higher densities of colonies have more potential for outbreeding and also have more potential to exchange microbiota. If this is true, then the relationship between genetic diversity and gut bacterial community diversity might be driven entirely by the environment. We believe the results of Matilla et al. (2018) make this explanation unlikely, but additional work will be needed to demonstrate this conclusively.

## CONCLUSION

This study provides a comprehensive analysis of spatial variation in honeybee gut bacterial community at the regional scale, and new evidence for the effects of land use and host genetic diversity on the gut microbiota. Given the large number of hypotheses we studied, we should expect at least some false positive results, and future work should seek to replicate the patterns we report. Our results provide the foundation for this and other work and advance our general understanding of the honeybee microbiota.

## AUTHOR CONTRIBUTIONS

J. K. Rowntree, Werner Müller, Latha Vellaniparambil and Calum Bridson conceived and designed the study; Latha Vellaniparambil recruited beekeepers and collected samples; Calum Bridson and Rachel E. Antwis performed the molecular laboratory work; Calum Bridson conducted bioinformatics; Calum Bridson, T. Tucker Gilman and J. K. Rowntree performed data analysis; Calum Bridson, T. Tucker Gilman and J. K. Rowntree wrote the manuscript. All authors gave final approval for publication.

## CONFLICT OF INTEREST

The author declares that there is no conflict of interest

## Supporting information


**Appendix S1** Supporting information

## Data Availability

Sequence data will be deposited in the NCBI SRA database, and metadata will be available from the Dryad Digital Repository upon acceptance. For reviewing purposes, Dryad data are available via: https://datadryad.org/stash/share/WILx7Pkgpawr36unMEtwJa7a3BUEDuEbfF1fMs_z2AE and sequencing data via: https://dataview.ncbi.nlm.nih.gov/object/PRJNA800821?reviewer=rffoos22fg6d52bgvpuo10fodf
