# Supplementary material for: Genetic diversity of honeybee colonies predicts gut bacterial diversity of individual colony members
Source: Environ Microbiol. 2022 Aug 12;24(12):5643–53. doi: 10.1111/1462-2920.16150 (PMC10087737; doi:10.1111/1462-2920.16150)
Supplement: Supplementary file 1 — Appendix S1 Supporting information [file EMI-24-5643-s001.docx]

**Supplementary Material**

**Genetic diversity of honeybee colonies predicts gut bacterial diversity of individual colony members**

# Bridson, C^1,4,6,7^; Vellaniparambil, L^2^; Antwis, R E^3^; Müller, W^4,5^; Gilman, R T^1^; Rowntree, J K^2^.

^1^Faculty of Science and Engineering, University of Manchester, Manchester, UK, M13 9PT

^2^Ecology and Environment Research Centre, Department of Natural Sciences, Manchester Metropolitan University, Manchester, UK, M1 5GD

^3^School of Science, Engineering and Environment, University of Salford, Salford, UK, M5 4WT

^4^Faculty of Biology Medicine and Health, Lydia Becker Institute of Immunology and Inflammation, University of Manchester, Manchester, UK, M13 9PT

^5^Miltenyi Biotec, Bergisch Gladbach, Germany

^6^Department of Infectious Diseases, Medical Microbiology and Hygiene, University of Heidelberg, Heidelberg, Germany

^7^Translational Lung Research Centre (TLRC), Heidelberg, Germany


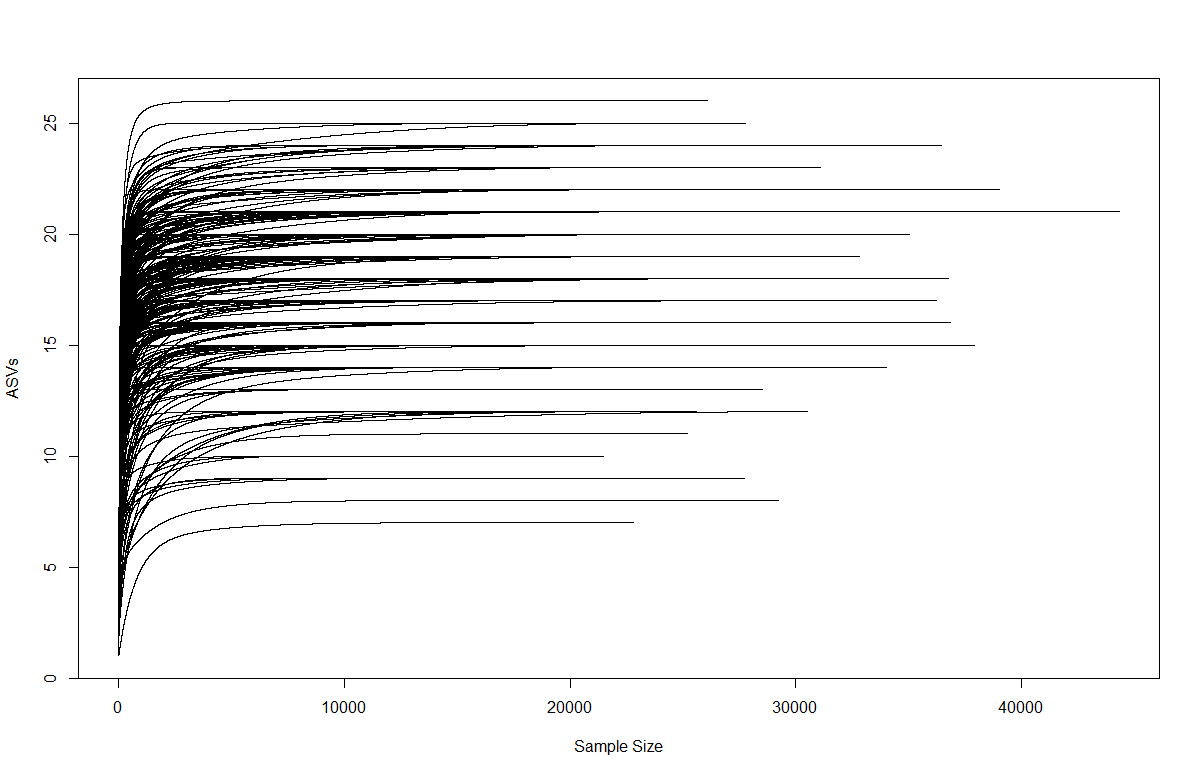


**Figure S1.** Rarefaction curves for each of the 293 honeybee gut samples sequenced using the 515F and 806R universal primers to amplify the V4 region of the 16S rRNA gene on the MiSeq platform (Illumina v2 chemistry).


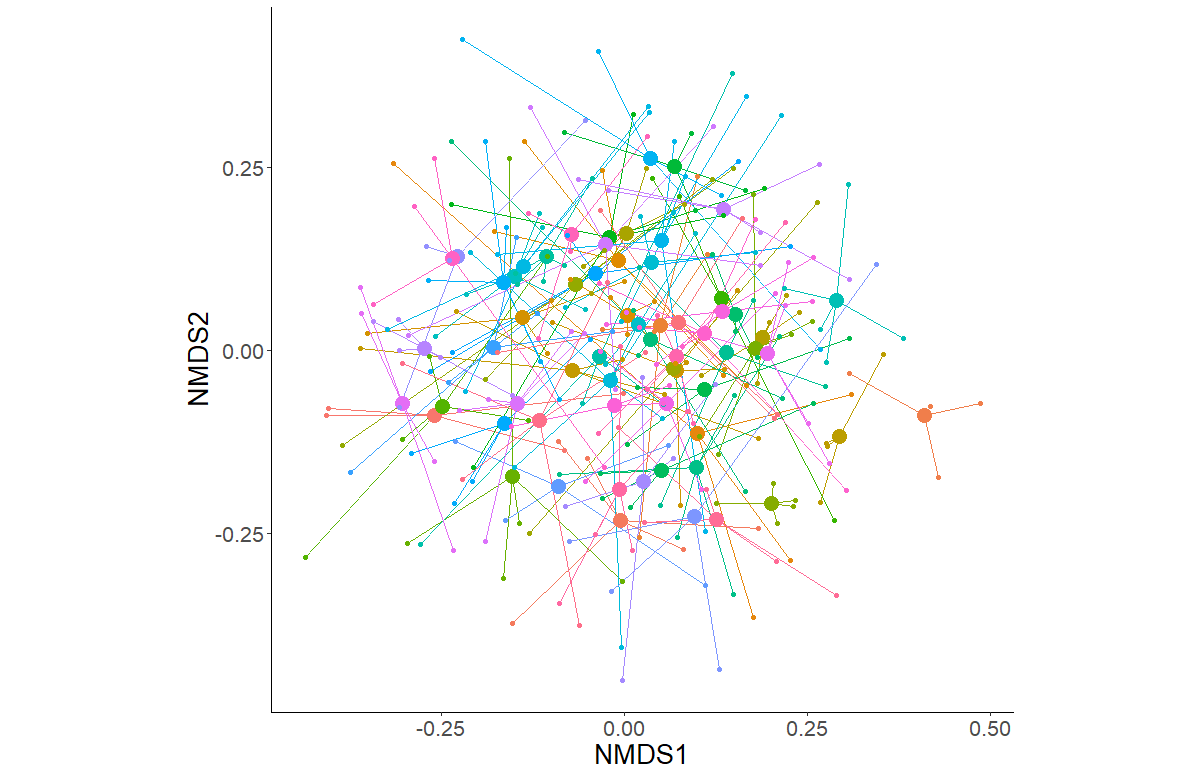


**Figure S2.** Non-metric Multidimensional Scaling (nMDS) ordination of Roger’s genetic distances among individual honeybees. Each small data point represents an individual bee, with lines connecting individuals to their colony centroid (larger circles).

**Table S1.** Summary of the abundances of the core gut bacterial taxa found from 63 hives in NW England including: the total number of reads of all ASVs associated with a species cluster; the total number of ASVs belonging to each core species cluster (Number of Variants); the percentage of reads of a species cluster that belong to the most abundant ASV of that cluster (% Most Abundant Variant); the percentage abundance of that species cluster in the region as a whole; and the percentage abundance of the species cluster within only the top 20 most abundant ASVs.

| **Taxa** | **Number of Reads** | **Number of Variants** | **% Most Abundant Variant** | **Overall Abundance (%)** | **Abundance in Top 20 ASVs (%)** |
| --- | --- | --- | --- | --- | --- |
| *Gilliamella* | 1383612 | 13 | 97.6 | 20.1 | 20.7 |
| *Frischella* | 479766 | 8 | 75.5 | 7.0 | 7.0 |
| *Snodgrassella* | 750352 | 5 | 77.4 | 10.9 | 11.5 |
| *Lactobacillus*  Firm 4 | 511096 | 14 | 37.5 | 7.4 | 6.9 |
| *Lactobacillus*  Firm 5 | 1965934 | 25 | 28.3 | 28.5 | 28.6 |
| *Bifidobacteria* | 832217 | 5 | 94.4 | 12.1 | 12.7 |
| *Bartonella* | 568797 | 3 | 93.9 | 8.3 | 8.2 |
| *Commensalibacter* | 266042 | 8 | 66.4 | 3.9 | 3.5 |

**Table S2.** The prevalence of each of the core species clusters across individual samples and for samples pooled at the colony level. Prevalence is reported as the percentage of individuals or colonies that possess at least one ASV from the species cluster.

| **Species Cluster** | **Presence in Individuals/ %** | **Presence in Colonies/ %** |
| --- | --- | --- |
| *Gilliamella* | 99.7 | 100 |
| *Frischella* | 92.5 | 100 |
| *Snodgrassella* | 98.3 | 100 |
| *Lactobacillus* Frim 4 | 93.9 | 100 |
| *Lactobacillus* Firm 5 | 100 | 100 |
| *Bifidobacteria* | 98.3 | 100 |
| *Bartonella* | 90.8 | 100 |
| *Commensalibacter* | 83.6 | 100 |

**Table S3.** Number of alleles, expected (H_e_) and observed (H_o_)Heterozygosity for each of the five microsatellite loci used for genotyping in this study.

| **Locus** | **Number of alleles** | **H_e_** | **H_o_** |
| --- | --- | --- | --- |
| A113 | 16 | 0.69 | 0.66 |
| AP043 | 9 | 0.76 | 0.70 |
| AP055 | 16 | 0.80 | 0.81 |
| A007 | 22 | 0.78 | 0.77 |
| B124 | 18 | 0.87 | 0.85 |

**Table S4.** The total number of alleles, individual Heterozygosity and mean Heterozygosity (H_s_) across all loci for each of the 63 honeybee colonies sampled in NW England.

|  |  | **Individual heterozygosity** | | | | |  |
| --- | --- | --- | --- | --- | --- | --- | --- |
| **Colony ID** | **Number of alleles** | **A113** | **AP043** | **AP055** | **A007** | **B124** | **H_s_** |
| ADW | 17 | 0.37 | 0.35 | 0.63 | 0.8 | 0.75 | 0.58 |
| AGE | 14 | 0.37 | 0.45 | 0.68 | 0.72 | 0.55 | 0.554 |
| AND | 14 | 0.71 | 0.25 | 0.58 | 0.25 | 0.67 | 0.492 |
| ANDZ | 15 | 0.6 | 0.5 | 0.6 | 0.68 | 0.75 | 0.626 |
| AR | 22 | 0.88 | 0.5 | 0.83 | 0.83 | 0.67 | 0.742 |
| ARZ | 22 | 0.68 | 0.8 | 0.72 | 0.75 | 0.82 | 0.754 |
| AT | 17 | 0.5 | 0.68 | 0.7 | 0.63 | 0.68 | 0.638 |
| BIC | 17 | 0.58 | 0.58 | 0.46 | 0.83 | 0.83 | 0.656 |
| BPO | 17 | 0.65 | 0.7 | 0.6 | 0.73 | 0.68 | 0.672 |
| CAT | 16 | 0.68 | 0.7 | 0.75 | 0.8 | 0.45 | 0.676 |
| CGH | 12 | 0.5 | 0.42 | 0.5 | 0.62 | 0.62 | 0.532 |
| CGR | 19 | 0.53 | 0.6 | 0.75 | 0.53 | 0.78 | 0.638 |
| CHA | 15 | 0.42 | 0.42 | 0.62 | 0.71 | 0.71 | 0.576 |
| CK | 14 | 0.75 | 0.75 | 0.75 | 0.75 | 0.5 | 0.7 |
| CWI | 15 | 0.6 | 0.78 | 0.75 | 0.35 | 0.68 | 0.632 |
| DEB | 14 | 0.8 | 0.35 | 0.65 | 0.5 | 0.6 | 0.58 |
| DEL | 15 | 0.35 | 0.4 | 0.75 | 0.5 | 0.68 | 0.536 |
| DH | 19 | 0.75 | 0.8 | 0.75 | 0.5 | 0.58 | 0.676 |
| DJ | 17 | 0.2 | 0.75 | 0.6 | 0.78 | 0.7 | 0.606 |
| DLY | 15 | 0.33 | 0.67 | 0.75 | 0.92 | 0.67 | 0.668 |
|  |  | **Individual heterozygosity** | | | | |  |
| **Colony** | **Number of alleles** | **A113** | **AP043** | **AP055** | **A007** | **B124** | **H_s_** |
| EAD | 18 | 0.5 | 0.6 | 0.78 | 0.72 | 0.75 | 0.67 |
| EFL | 16 | 0.46 | 0.46 | 0.75 | 0.71 | 0.62 | 0.6 |
| GB | 14 | 0.5 | 0.58 | 0.5 | 0.5 | 0.92 | 0.6 |
| HAR | 16 | 0.75 | 0.67 | 0.75 | 0.42 | 0.79 | 0.676 |
| HMO | 12 | 0.67 | 0.75 | 0.5 | 0 | 0.67 | 0.518 |
| HSS | 18 | 0.7 | 0.55 | 0.65 | 0.7 | 0.79 | 0.678 |
| IL | 16 | 0.8 | 0.65 | 0.5 | 0.7 | 0.68 | 0.666 |
| IMO | 17 | 0.65 | 0.68 | 0.68 | 0.53 | 0.78 | 0.664 |
| IRLO | 15 | 0.5 | 0.5 | 0.62 | 0.7 | 0.75 | 0.614 |
| JA | 14 | 0.67 | 0.71 | 0.25 | 0.67 | 0.67 | 0.594 |
| JCH | 15 | 0.2 | 0.65 | 0.62 | 0.37 | 0.75 | 0.518 |
| JENE | 18 | 0.58 | 0.53 | 0.7 | 0.72 | 0.65 | 0.636 |
| JGPH | 15 | 0.5 | 0.75 | 0.58 | 0.67 | 0.75 | 0.65 |
| JHW | 17 | 0.45 | 0.65 | 0.65 | 0.75 | 0.73 | 0.646 |
| JL | 15 | 0.7 | 0.55 | 0.35 | 0.85 | 0.65 | 0.62 |
| JN | 16 | 0.6 | 0.5 | 0.35 | 0.7 | 0.8 | 0.59 |
| KIN | 20 | 0.53 | 0.78 | 0.75 | 0.72 | 0.72 | 0.7 |
| KK | 16 | 0.42 | 0.62 | 0.46 | 0.71 | 0.62 | 0.566 |
| KTH | 20 | 0.82 | 0.7 | 0.72 | 0.75 | 0.63 | 0.724 |
| LIS | 17 | 0.65 | 0.6 | 0.6 | 0.75 | 0.7 | 0.66 |
| LWA | 17 | 0.6 | 0.75 | 0.45 | 0.63 | 0.8 | 0.646 |
| MAD | 17 | 0.45 | 0.8 | 0.68 | 0.62 | 0.55 | 0.62 |
| MAN | 19 | 0.55 | 0.6 | 0.8 | 0.68 | 0.82 | 0.69 |
|  |  | **Individual heterozygosity** | | | | |  |
| **Colony** | **Number of alleles** | **A113** | **AP043** | **AP055** | **A007** | **B124** | **H_s_** |
| MB | 16 | 0.79 | 0.67 | 0.42 | 0.79 | 0.62 | 0.658 |
| MPO | 13 | 0.55 | 0.6 | 0.45 | 0.5 | 0.58 | 0.536 |
| MST | 20 | 0.82 | 0.7 | 0.75 | 0.62 | 0.75 | 0.728 |
| ND | 13 | 0 | 0.35 | 0.6 | 0.6 | 0.8 | 0.47 |
| NW | 18 | 0.37 | 0.68 | 0.78 | 0.6 | 0.78 | 0.642 |
| PBO | 18 | 0.62 | 0.7 | 0.4 | 0.75 | 0.85 | 0.664 |
| PGS | 10 | 0.35 | 0.5 | 0.5 | 0.5 | 0.35 | 0.44 |
| PL | 15 | 0 | 0.71 | 0.67 | 0.58 | 0.75 | 0.542 |
| PMA | 19 | 0.5 | 0.63 | 0.72 | 0.68 | 0.85 | 0.676 |
| REN | 15 | 0.71 | 0.5 | 0.67 | 0.42 | 0.88 | 0.636 |
| RW | 17 | 0.58 | 0.62 | 0.71 | 0.71 | 0.83 | 0.69 |
| SH | 15 | 0.67 | 0.58 | 0.67 | 0.67 | 0.67 | 0.652 |
| SIW | 18 | 0.58 | 0.68 | 0.7 | 0.78 | 0.75 | 0.698 |
| SSP | 19 | 0.78 | 0.65 | 0.8 | 0.6 | 0.68 | 0.702 |
| TAY | 15 | 0.67 | 0.67 | 0.75 | 0.58 | 0.58 | 0.65 |
| TEM | 17 | 0.5 | 0.78 | 0.7 | 0.6 | 0.72 | 0.66 |
| TSE | 23 | 0.8 | 0.85 | 0.78 | 0.72 | 0.88 | 0.806 |
| VCU | 19 | 0.7 | 0.45 | 0.58 | 0.75 | 0.72 | 0.64 |
| VHA | 22 | 0.5 | 0.78 | 0.63 | 0.82 | 0.82 | 0.71 |
| WIL | 21 | 0.8 | 0.68 | 0.8 | 0.53 | 0.88 | 0.738 |

**Table S5.** Factors predicting alpha diversity at the colony level. For each Hill number (q), the first number is the effect size scaled to the standard deviation of the predictor in the population, and the second number is the p-value associated with the predicter. • indicates a marginally significant effect. The effect size is the change in Hill number that we would expect for a change of one standard deviation in the predictor. Positive (negative) effects indicate that alpha diversity increases (decreases) as the predictor increases.

|  | **q = 0** | | **q = 1** | | **q = 2** | |
| --- | --- | --- | --- | --- | --- | --- |
|  | **effect** | **p-value** | **effect** | **p-value** | **effect** | **p-value** |
| proportion of alleles shared between pairs of individuals | -0.33 | 0.486 | -0.51 | 0.095^•^ | -0.436 | 0.102 |
| mean heterozygosity | 0.53 | 0.266 | -0.06 | 0.852 | -0.10 | 0.686 |
| landscape diversity | -0.13 | 0.792 | 0.09 | 0.867 | 0.12 | 0.741 |
| proportion of urban land | -0.92 | 0.062^•^ | -0.37 | 0.274 | -0.20 | 0.477 |
| northing | -0.22 | 0.667 | -0.23 | 0.496 | -0.13 | 0.642 |
| easting | 0.49 | 0.364 | 0.47 | 0.170 | 0.30 | 0.322 |

**Table S6.** Factors predicting alpha diversity at the individual level. For each Hill number (q), the first number is the effect size scaled to the standard deviation of the predictor in the population, and the second number is the p-value associated with the predictor. * indicates a significant effect. The effect size is the change in the Hill number that we would expect for a change of one standard deviation in the predictor. Positive (negative) effects indicate that alpha diversity increases (decreases) as the predictor increases.

|  | **q = 0** | | **q = 1** | | **q = 2** | |
| --- | --- | --- | --- | --- | --- | --- |
|  | **effect** | **p-value** | **effect** | **p-value** | **effect** | **p-value** |
| proportion of alleles shared between pairs of individuals | -0.33 | 0.251 | -0.47 | 0.040* | -0.38 | 0.040* |
| heterozygosity (individual) | 0.04 | 0.855 | -0.12 | 0.454 | -0.11 | 0.395 |
| mean heterozygosity (colony) | -0.04 | 0.902 | -0.14 | 0.554 | -0.09 | 0.650 |
| landscape diversity | -0.20 | 0.556 | 0.13 | 0.661 | 0.16 | 0.483 |
| proportion of urban land | -0.32 | 0.300 | -0.24 | 0.312 | -0.19 | 0.311 |
| northing | 0.39 | 0.204 | 0.15 | 0.532 | 0.05 | 0.797 |
| easting | 0.25 | 0.446 | 0.19 | 0.473 | 0.17 | 0.451 |

**Table S7.** Primer pairs used to amplify the five microsatellite loci (from Evans *et al.* 2013).

| **Primer Name** | **Sequence** |
| --- | --- |
| A113-F-(FAM) | CTC GAA TCG TGG CGT CC |
| A113-R | CCT GTA TTT TGC AAC CTC GC |
| A007-F-(TAMRA) | GTT AGT GCC CTC CTC TTG C |
| A007-R | CCC TTC CTC TTT CAT CTT CC |
| AP043-F-(JOE) | GGC GTG CAC AGC TTA TTC C |
| AP043-R | CGA AGG TGG TTT CAG GCC |
| AP055-F-(ROX) | GAT CAC TTC GTT TCA ACC GT |
| AP055-R | CAT TCG GTA TGG TAC GAC CT |
| B124-F-(FAM) | GCA ACA GGT CGG GTT AGA G |
| B124-R | CAG GAT AGG GTA GGT AAG CAG |

Table S8. The relationship between pooled colony microbiota diversity (estimated using Hill numbers when q=0, 1 and 2) and the proportion of particular land use types around the colony. Only the land use types that make up >0.1% of the area around the colony in >30% of colonies are included. The relationship was determined using the information theoretic approach outlined in the main methods for predicting colony microbiota diversity from genetic and landscape factors. The analysis for each land use type was performed independently. The effect sizes and p-values represent the same as in Table S5 and Table S6. We did not correct for multiple tests, and a lot of tests were carried out, which reduces our confidence in the results, but they are provided to allow researchers to compare the trends to patterns in their own data.

|  | **q=0** | | **q=1** | | **q=2** | |
| --- | --- | --- | --- | --- | --- | --- |
| **Land Use Type** | **effect** | **p-value** | **effect** | **p-value** | **effect** | **p-value** |
| Urban | -0.92 | 0.062 | -0.37 | 0.274 | -0.20 | 0.477 |
| Suburban | -0.63 | 0.199 | -0.15 | 0.658 | -0.07 | 0.794 |
| Arable | 0.02 | 0.978 | 0.21 | 0.604 | 0.15 | 0.659 |
| Improved Grassland | 1.04 | 0.036 | 0.11 | 0.748 | 0.02 | 0.953 |
| Neutral Grassland | 0.60 | 0.205 | 0.43 | 0.163 | 0.36 | 0.181 |
| Broadleaved Forest | -0.66 | 0.190 | 0.19 | 0.557 | 0.28 | 0.309 |
| Littoral Sediment | -0.01 | 0.982 | -0.39 | 0.269 | -0.33 | 0.294 |
| Supra-Littoral Sediment | 0.40 | 0.464 | -0.09 | 0.798 | -0.06 | 0.857 |
| Inland Rock | 0.24 | 0.701 | 0.43 | 0.158 | 0.28 | 0.281 |
| Saltwater | 0.38 | 0.845 | -0.38 | 0.230 | -0.28 | 0.317 |
